# How can we improve crop genotypes to increase stress resilience and productivity in a future climate? A new crop screening method based on productivity and resistance to abiotic stress

**DOI:** 10.1093/jxb/erw330

**Published:** 2016-09-27

**Authors:** Arnauld A. Thiry, Perla N. Chavez Dulanto, Matthew P. Reynolds, William J. Davies

**Affiliations:** ^1^International Maize and Wheat Improvement Centre (CIMMYT), Crrtra. Mexico-Veracruz km 45, Col. El Batan, Texcoco, Edo. de Mexico, CP 56130, Mexico; ^2^The Lancaster Environment Centre, Lancaster University, Bailrigg, Lancaster LA1 4YQ, UK

**Keywords:** Abiotic stress indices, bread wheat, crop breeding, drought tolerance index, productivity, resilience.

## Abstract

The need to accelerate the selection of crop genotypes that are both resistant to and productive under abiotic stress is enhanced by global warming and the increase in demand for food by a growing world population. In this paper, we propose a new method for evaluation of wheat genotypes in terms of their resilience to stress and their production capacity. The method quantifies the components of a new index related to yield under abiotic stress based on previously developed stress indices, namely the stress susceptibility index, the stress tolerance index, the mean production index, the geometric mean production index, and the tolerance index, which were created originally to evaluate drought adaptation. The method, based on a scoring scale, offers simple and easy visualization and identification of resilient, productive and/or contrasting genotypes according to grain yield. This new selection method could help breeders and researchers by defining clear and strong criteria to identify genotypes with high resilience and high productivity and provide a clear visualization of contrasts in terms of grain yield production under stress. It is also expected that this methodology will reduce the time required for first selection and the number of first-selected genotypes for further evaluation by breeders and provide a basis for appropriate comparisons of genotypes that would help reveal the biology behind high stress productivity of crops.

## Highlight

A new scoring scale based on previously developed stress indices generated a new stress index for crop screening, providing simple and easy identification of yield-resilient, productive and/or contrasting genotypes.

## Introduction

In agriculture, drought is by far the most important environmental stress that constrains crop yield (Blum, 2011). More than 40% of the world is classified as dry land, of which 8% is subhumid and 16% is semiarid (Pretty *et al.*, 2005; Middleton *et al.*, 2011). In addition, increasing temperature is an important component of climate change and its negative impact on yield is expected to increase in the future. Indeed, it has been demonstrated that growing wheat crops under heat stress (day/night temperatures 30/25 °C) can lead to a 30–35% reduction in yield grain weight, when compared with controls (18/13 °C) (Wardlaw *et al.*1989), and the importance of incorporating a heat tolerance trait into wheat germplasm has been highlighted (Sareen *et al.* 2012). We need to develop genotypes with the capacity to yield significantly under heat stressed environments (Sareen *et al.* 2012). Therefore, understanding more about the mechanisms involved in plant tolerance/resistance to high temperature and drought stress becomes key for future improved crop production under stress as the climate in many food producing regions becomes hotter and drier (Blum, 2011; Macková *et al.*, 2013).

Many efforts have been made to improve crop productivity under water-limiting conditions. While breeding activity has directed selection towards increasing the economic yield of cultivated species, natural selection has favoured mechanisms of adaptation and survival (Cattivelli *et al.*, 2008). More than 80 years of breeding activities have focused on the increase of yield under drought environments for different crop plants. Meanwhile, significant gains in understanding the physiological and molecular responses of plants to water deficits have been provided by fundamental research (Cattivelli *et al.*, 2008).

However, in both conventional breeding and biotechnology the drought-resistant ideotype is not always well defined and traits that might deliver high drought productivity are not always clear (Blum, 2005). Further, we have made little progress in identifying key mechanisms involved in delivering a combination of high productivity and stress resilience.

There is a need to define and properly characterize what it is meant by the term ‘stress tolerant genotype’. The concepts of drought tolerance as set out in the literature can differ significantly. Effectively, the ecological definition of drought resistance is the ability to stay alive during periods of low water supply (Levitt *et al.*, 1960 cited in Turner, 1979). Alternatively, for crop species, drought tolerance is defined as the ability of plants to grow and reproduce satisfactorily to produce harvestable yield with limited water supply or when under periodic water deficit (Turner, 1979; Fleury *et al.*, 2010). It has been suggested that yield stability is a better indicator of genotypic drought resistance compared with grain yield under stress (Blum *et al.*, 1989). In terms of physiological mechanism, drought resistance is often considered as a compromise between ‘dehydration avoidance’ and ‘dehydration tolerance’, both of which can have variable impacts on yield (Fischer and Maurer, 1978; Turner, 1979, 1986; Levitt, 1980). Additionally, the concept of escape strategy mentioned by Turner (1979) and Levitt (1980) includes phenological development speed as a criterion for selection, in order to avoid selecting early genotypes within a population that also contains genotypes with a longer phenological development under stress.

Plant breeding programmes mainly focus on selecting genotypes that have high yield firstly under yield potential conditions (non-stress) and secondly under stress conditions. To reach this aim, the classical postulate, widely accepted by breeders for selection, is that a genotype with high yield potential will perform well under most environments (Blum, 2005). However, this selection method does not include the concept of yield stability and neither does it consider adaptation to a stress environment. Such shortcomings can be a cause of slow progress in breeding (Ceccarelli and Grando, 1991; Blum, 1996).

Several stress indices, described in Supplementary Appendix A at *JXB* online, have been proposed to allow screening for drought stress adaptation. Fisher & Maurer (1978) developed a stress susceptibility index (SSI), Rosielle & Hamblin (1981) defined the tolerance index (TOL) and the mean productivity index (MP), and Fernandez (1992) analysed the latter and created two new indices, the geometric mean productivity index (GMP) and the stress tolerance index (STI) in an attempt to improve the MP so that it would identify highly productive genotypes in both stress and non-stress environments. These various indices consider the relationships between traits, in non-stress (yield potential, irrigated conditions) and stress (mainly drought) environments. These indices were grouped into two classes, according to Rosielle and Hamblin (1981), Fernandez (1992) and Sareen *et al.* (2012). The first represents the susceptibility indices (SSI and TOL) which tend to distinguish between the stress-tolerant and the stress-susceptible genotypes, showing a negative relationship with yield. The second class represents the tolerance indices (MP, GMP and particularly STI) that tend to identify genotypes with stress tolerance and high average yield, showing a positive relationship with yield. However, tolerance and susceptibility indices are not ideal to characterize genotypes with high yield performance and high stress tolerance under both environments. Genotype yield performance under stress and non-stress conditions has been categorized by Fernandez (1992) into four groups: A: genotypes expressing uniform superiority in both stress and non-stress conditions; B: genotypes expressing good performance only in yield potential conditions and not under stress conditions; C: genotypes presenting a relatively higher yield only under stress; D: genotypes with poor yield performance in both environments. Additionally, Fernandez (1992) evidenced some failures of the defined indices to distinguish between certain of these groups and suggested that STI is generally able to distinguish better group A from groups B and C (Table 1).

**Table 1.** Summary of the interpretation of tolerances according to the previously developed indices (stress susceptibility index (SSI), tolerance index (TOL), mean productivity index (MP), geometric mean productivity index (GMP), and stress tolerance index (STI)) and their failures to distinguish the different response groups of plants defined by Fernandez (1992) Classes 1 and 2 correspond to susceptibility indices and tolerance indices, respectively, where class 1 tends to distinguish between the stress-tolerant and the stress-susceptible genotypes and class 2 tends to identify genotypes with stress tolerance and high average yield. Group A genotypes express uniform superiority in both stress and non-stress condition; group B genotypes express good performance only in yield potential but not under stress conditions; group C genotypes present a relatively higher yield only under stress; group D genotypes have poor yield performance in both environments.

Index	Index value	Tolerance	Fails	Class
SSI	High	Low	Fails in distinguishing A and C	1
TOL	High	Low	Fails in distinguishing A and C	1
MP	High	High	Fails in distinguishing A and B	2
GMP	High	High	Same failure as MP, but distinguishes A better than MP	2
STI	High	High	Same failure as MP but distinguishes A better than MP and GMP	2

There is a clear need to develop an accurate tool able to identify the yield performance and resilience capacity of genotypes under stress conditions, since previous research has focused only on yield performance without taking resilience or stability into account. Currently, STI, GMP and MP are the most recommended indices to identify genotypes with high yield in both non-stress and stress environments (heat and drought) (Khodarahmpour *et al.*, 2011; Mohammadi *et al.*, 2011; Sareen *et al.*, 2012). In contrast, Khayatnezhad *et al.*(2010) stated that none of these indices could clearly identify cultivars with high yield in both environments (stress and non-stress).

Importantly, there is not yet an accurate screening index that can be recommended in breeding programmes to select genotypes for abiotic stress adaptation and high yield in both stress and non-stress environments. However, it has been suggested that a combination of stress indices (tolerance and suceptibility indices) might provide a more useful criterion for improving drought stress tolerance selection in common bean and heat stress tolerance selection in maize (Ramirez-Vallejo and Kelly, 1998; Khodarahmpour *et al.*, 2011). Nevertheless, it is not yet clear how to combine stress indices appropriately.

Therefore, the main objective of the present work was to develop a new simple tool based on the complementarities of two classes of indices (class 1, susceptibility indices; and class 2, tolerance indices; see Table 1) to express crop yield, in order to elucidate the characteristics of the best performing and adapted genotypes under stress. To achieve this goal, we developed a methodology to enable us to combine indices. We suggest how this tool can be used in crop breeding programmes and show how the new indices can be used to provide a focus for mechanistic research aimed at understanding the basis of the sensitivity of crop yield to environmental stresses.

## Materials and methods

### Site of experiments

Field trials were conducted at the Mexican Phenotyping Platform (MEXPLAT), located in the highly productive irrigated spring wheat growing environment in the Yaqui Valley, near Obregon City, NW Mexico (27° 22′ 6.9″ N, 109° 55′ 21.6″ W, 38 m above sea level). This site is a temperate high radiation environment, and with adequate irrigation average yield of the best lines is approximately 8 t ha^–1^ (Sayre *et al.*, 1997).

### Experimental material and stress treatments

The CIMCOG-ROOT trial was used in this study. It consists of ten lines selected from the CIMMYT Core Germplasm (CIMCOG) trial representing contrasting genotypes for partitioning and related traits. These wheat lines were evaluated during two cropping seasons, 2012–13 and 2013–14, in three different environments: irrigated conditions (yield potential) for the two cropping seasons (from November to early May), and under drought and irrigated heat stress during the later cropping season, i.e. from December 2013 to late May 2014, and from February to June 2013, respectively. All trials were conducted with optimal crop management following a preventive biotic stress control strategy in order to control other stresses and with conventional nutrients supplied.

For all the experiments, the testing area was surrounded by durum wheat (*Triticum durum*) that acted as a windbreak to reduce edge effects. The experimental design was a total randomized block with three replications for the grain yield under yield potential conditions (Yp) and drought trials, and two replications for the heat trial. Irrigation was gravity-fed flood applied for all experiments. For the drought stress trial, the last irrigation was at 50% of seedling emergence, and in the case of the Yp and irrigated heat trial, four additional irrigations were applied, after 50% of emergence, every 3 weeks until 15 days before maturity.

Additionally, data from a set of 294 elite genotypes—the Wheat Association Mapping Initiative (WAMI) trial—grown under yield potential conditions during the 2011–2012 cycle (November to May) and under heat conditions from February to June 2012 have been used to test the robustness of the new indices.

### Selection of stress-adapted genotypes within a population under field conditions

The selection of crop genotypes under field conditions presents difficulties due to the variability, intensity, timing and duration of the abiotic stress (heat and/or drought), as well as the development of several stresses at the same time (e.g. pest invasion and nutrient stress). Therefore, it is important to compare genotypes within the population response in order to identify those that are more or less susceptible and/or tolerant to the stress in question. Screening for stress-adapted genotypes under field conditions is made through the susceptibility to accumulative stress and the interaction between responses. Consequently, to study a specific mechanistic response to environmental stress by plants under field conditions it is highly important to control as much as possible the other collateral stresses that can appear during a growing season (biotic stress, nutrient stress, etc.) in order to reduce their effects on the crops (Chapin *et al.*, 1987; Herms and Mattson, 1992; Dolferus *et al.*, 2011). However, the control will never completely reduce the pressure of the other stresses, and so we assume that a population of genotypes grown during the same season would have suffered a similar pressure of cumulative stress (abiotic and biotic stress). Therefore, we suggest that each genotype should be compared within the population response each season to better understand the stress adaptation.

### Basis of the development of the new stress indices and their uses

As mentioned above, different approaches are used to identify tolerant genotypes for the indices from classes 1 and 2 (Table 1), as class 1 tends to discriminate the tolerant from the susceptible, and class 2 tends to distinguish the tolerant with high mean yield. However, the failures of the indices in identifying the best yielding genotypes under stress (Table 1) show that a high yield under non-stress conditions does not automatically indicate a good performance under stress, and similarly, a high yield under stress does not automatically indicate high resilience. The outcome of a stress challenge will depend on the severity of the stress and obviously on the characteristics of the genotype (genetic effects).

Nevertheless, both classes of indices (classes 1 and 2, susceptibility and tolerance indices, respectively; Table 1) explain a part of the behaviour of the genotypes under stress. Therefore, based on the previous concept developed by Fernandez (1992), Fisher & Maurer (1978) and Rosielle & Hamblin (1981), we propose here two new indices that are compiled through the combination of the score indices (described below) that show a high correlation with yield in stress and non-stress environments. The score indices have been classified within two new scales called resilience and production capacity, based on classes 1 and 2 of the existing stress indices (Table 1), respectively.

The new resilience and production capacity indices are defined as follows: (i) the resilience capacity index (RCI) expresses the yield decrease of the genotypes under stress within a population, compared with yield potential conditions, and (ii) the production capacity index (PCI) expresses the mean production of the genotypes under both stressed and non-stressed conditions within a population. These indices constitute an attempt to improve the use of the five previous indices (SSI, TOL, MP, GMP and STI), as both new indices (RCI and PCI) are required if we are to understand the basis of any yield limitations under stress. Indeed, resilience, productivity and stability show a complex relationship in ecosystems (Xu and Li, 2002), such as a crop system under a specific environment, and we propose that these are all key issues to increase future crop production.

### Why combine the indices?

**Fig. 1.**
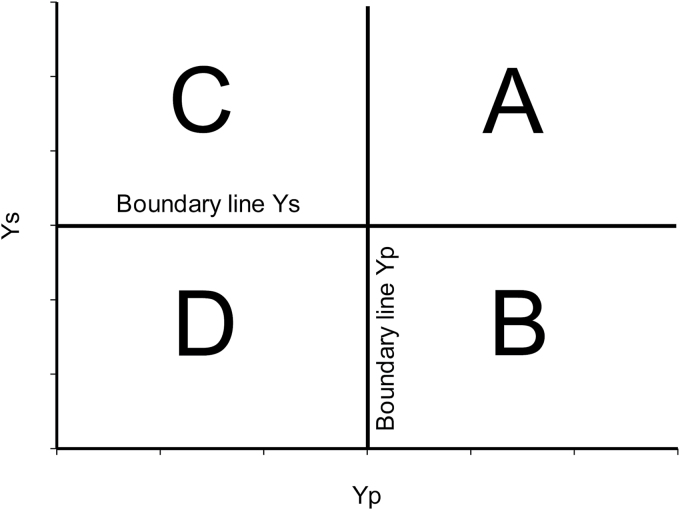
Representation of the different response groups (A, B, C, and D), defined by Fernandez (1992), according to their grain yield under abiotic stress conditions (Ys) and grain yield under yield potential conditions (Yp). Group A genotypes express uniform superiority in both stress and non-stress condition; group B genotypes express good performance only in yield potential and not under stress conditions; group C genotypes present a relatively higher yield only under stress; and group D genotypes express poor yield performance in both environments. The boundary lines (bold line) create the limit between one group and the others. The boundary line value corresponds to the yield value from a check under yield potential conditions (boundary line Yp) and yield under drought stress (boundary line Ys).

It is important to analyse the different groups of yield responses (from A to D). Groups A and D represent the extremes—in terms of grain yield—as the best and worst genotypes. However, extreme responses are rare and genotypes in these two groups would tend to group with B or C, such as AB or AC and DB or DC. Nevertheless, this could explain why both classes of indices have a relatively good relationship with both yields (non-stress and stress), as shown by Fernandez (1992), as they both fail to correctly identify the middle index values of the linear regression with yield (non-stress and stress). In turn, the middle values can have two tendencies, a medium-high or a medium-low value, for both environments. For example, group A has a value close to the boundary line that distinguishes group A from group C under non-stress and from group B under stress conditions (Fig. 1). Indeed, to distinguish these values that are more A than C under non-stress and vice versa, we will use the terms medium-high and medium-low, respectively. Considering this, medium values in the linear regression obtained with the indices have to be readjusted in order to express better the yield trait under non-stress and stress environments. This can be achieved by combining the indices.

### How can the indices be combined, as their values are totally different?

In order to classify a trait (e.g. tolerance) from the highest to the lowest, the indices (SSI, TOL, MP, GMP and STI) are each given their own numerical value, as individual index values can only be interpreted inside each index itself, because the scale or reference of the different indices is not the same. Additionally, indices of class 1 have a reverse scale from those of class 2, where low values mean high tolerance. Therefore, to enable comparison of the different indices, a scale has been created on the basis of an equal reference for all indices by scoring the results from 1 to 10, where a high value means always a good response in terms of resilience or production capacity. Afterwards, the five indices show a value for each genotype that is comparable between the different indices. The idea of scoring is to have an easy visualization of the information given by the indices for the population under study, and to be able to compare one index with the others. A whole number, on a 1 to 10 scale, provides an easier interpretation than decimal values allocated to the original equations. Additionally, it opens new insights by permitting arithmetic operations between the indices in a simple way.

### How can the scoring scale be created?

The scoring scale for each index is calculated on the global response within the overall population under study. Thus, the scale is adjusted with the minimum and maximum value obtained with the original equation of the index. The difference of these two values gives the range of the scale for each index. This range is divided into ten parts and each part has a score from 1 to 10. Therefore, each part represents 10%, 20%, ..., or 100% of the range value.

Additionally, we have inverted the value of TOL and SSI, so a high value obtained with the original equation will receive a lower score. This allows the two classes of indices to have the same scale, where a high score will always mean a ‘good’ genotype. For example, a score value of 2 is obtained in the different indices for all the values within 10–20% of the range for MP, GMP and STI and 80–90% of the range for TOL and SSI. A tool developed in Microsoft Excel has been created to assign a score to each value.

Once the scores have been obtained, we can easily combine and test them against yield under stress and non-stress conditions, and figure out if genotypes are better adapted to express yield under stress and/or non-stress by identifying or distinguishing better groups B and C based on the RCI and PCI. These are terms that indicate much more specifically what the indices are showing.

## Results and discussion

### Testing the methodology and the score indices

Firstly, the score indices were tested against their original value from each index. Table 2 shows the Pearson correlation coefficient between the score indices and the original indices calculated on yield data from the WAMI trial (294 genotypes). The Pearson correlation coefficient between the score stress susceptibility index (SSIs) and the score tolerance index (TOLs) values and their original index values (SSI and TOL) is highly negative (ranging from −0.78 to −0.98), as the score scale has been inverted in order to create a scale showing resilience instead of susceptibility. On the other hand, the Pearson correlation coefficients between the original values for MP, GMP and STI and the score indices MPs, GMPs and STIs are highly significant. These high Pearson correlation coefficient values demonstrate that the score indices can be used as a surrogate of their original index value.

**Table 2.** Pearson correlation coefficient between the score indices defined in this paper (SSIs, TOLs, MPs, GMPs and STIs) and their original indices (SSI, TOL, MP, GMP and STI) defined by the original authors, calculated on yield data from the WAMI trial (294 genotypes) SSI and TOL show a negative correlation with SSIs and TOLs, respectively, as the score scale has been inverted. *P<0.05.

	SSI	TOL	MP	GMP	STI
Class 1					
SSIs	−0.98*	−0.80*	0.07	0.38	0.38
TOLs	−0.78*	−0.97*	−0.51	−0.21	−0.21
Class 2					
MPs	−0.09	0.49	0.98*	0.93*	0.93*
GMPs	−0.40	0.19	0.93*	0.98*	0.98*
STIs	−0.39	0.19	0.93*	0.98*	0.99*

### How can the score indices be combined? What is the best combination?

**Fig. 2.**
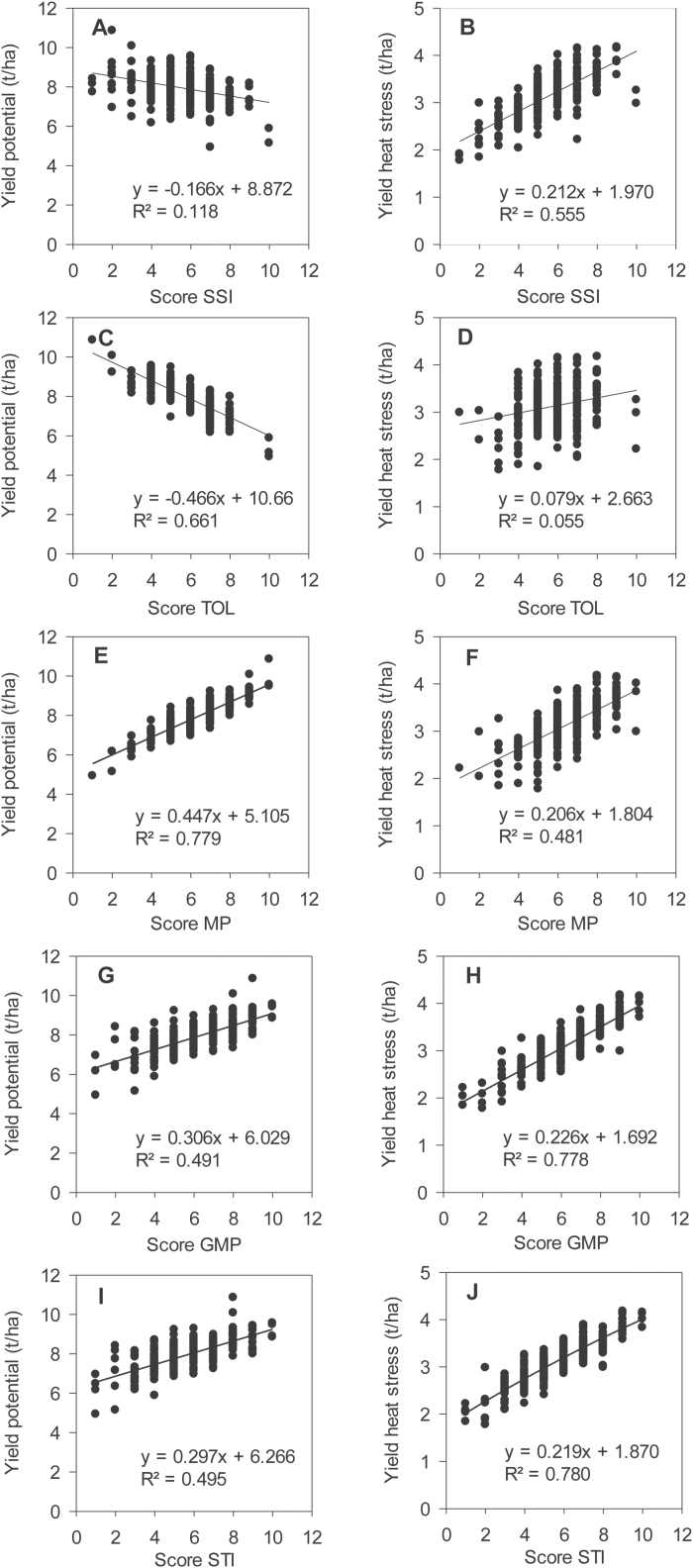
Linear regression and the coefficient of determination of the different score indices *versus* grain yield under yield potential and heat stress conditions. Calculations use yield data from the WAMI trial (294 genotypes) under heat stress and non-stress conditions during the cropping season 2011–2012.

Figure 2 shows the linear regression and the coefficient of determination of the different score indices *vs* yield under non-stress and heat stress environments, calculated on 294 genotypes from the WAMI trial. It shows that no index, used individually, could clearly identify the high yielding genotypes, independently of the environment. This result confirms the conclusion of Khayatnezhad *et al.* (2010) from a study with 22 genotypes of durum wheat. In each class of index (susceptibility and tolerance), SSI and STI show the closest relationship with yield under heat stress. In contrast, TOL and MP show a close relationship with yield potential. These responses would suggest that the combination of the score indices from each class would improve the relationship between the indices *per se* and grain yield.

### New indices

The new indices are based on the combination of score indices. In order to show and illustrate easily the score indices value and the contrast within the whole population, data from a smaller trial of ten genotypes from the CIMCOG-ROOT trial have been used to make the visualization easier (compared with a table with 294 genotypes). Nevertheless, the method to interpret and use the score is the same for ten, 294, or even more genotypes.

Table 3 is an example of a score index table using grain yield data of ten genotypes from the CIMCOG-ROOT trial. The Yp data used in this table are a mean for each genotype from two cropping seasons (2012–2013 and 2013–2014), in order to provide more consistent information on the yield potential of the genotype, considering that the Yp represents the maximum grain yield that a genotype is able to produce. Indeed, the ten score indices provide an illustration of small differences between SSI and TOL. On the other hand, GMP and STI were very similar, but both were slightly different from MP. Table 2 indicates also the correlation between the indices within each class of indices in the 294 genotypes trial. It is important to observe that in both cases the values are generally of the same magnitude within the two classes. Thus, these score values (Table 3) and the Pearson correlation coefficient (Table 2) confirm that SSI and TOL, and MP, GMP and STI, can be associated to class 1 and class 2, respectively (Table 1), demonstrating that these classes address two different characteristics, resilience capacity (RC) and production capacity (PC), respectively.

**Table 3.**
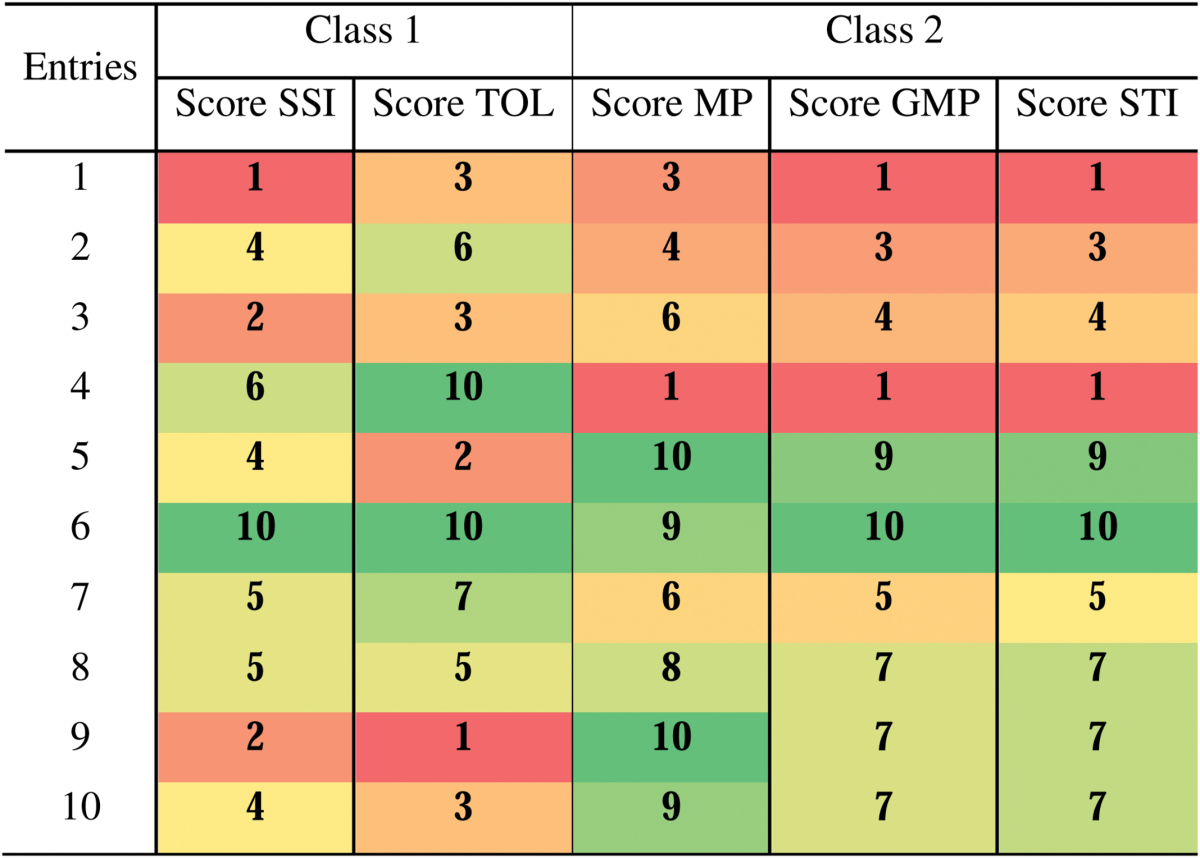
Example of a score index table based on grain yield data from the CIMCOG-ROOT trial (ten genotypes) under yield potential and heat stress environments, for the stress susceptibility index (SSI), tolerance index (TOL), mean productivity index (MP), geometric mean productivity index (GMP), and stress tolerance index (STI) during the 2012–13 cropping season The score indices show slight score differences but keep the same magnitude into each class, where class 1 tends to distinguish between the stress-tolerant and the stress-susceptible genotypes and class 2 tends to identify genotypes with stress tolerance and high average yield.

At this point, we asked which combination of these score indices could be considered as the best index to express yield under stress and non-stress conditions. Several combinations have been studied in order to generate a new index with the two components (RC and PC). The method used and the different combinations and formula are shown in Supplementary Table S1. The combinations taken into account, for each case, were achieved by pairs or groups of four score indices (combinations of two by two score indices from classes 1 and 2), adding or subtracting components. Each combination was correlated with grain yield under abiotic stress conditions (Ys) and grain yield under non-stress conditions (Yp) by calculating the Pearson correlation coefficient (Supplementary Tables S2–S4). Some combinations show a better correlation with Ys and Yp than others, and two of them are outstanding.

The highest Pearson correlation coefficient with Ys and with Yp for the three trials (CIMCOG-ROOT under drought and irrigated heat, and WAMI) will be referred to as yield stress score index (YSSI) and the yield potential score index (YPSI), respectively:

$$\text{YSSI}=\frac{\left(\text{STIs}+\text{SSIs}\right)}{2}$$ (1)

$$\text{YPSI}=\left(\frac{\left(\text{MPs+STIs}\right)}{2}-\frac{\left(\text{SSIs+TOLs}\right)}{2}\right)$$ (2)

**Fig. 3.**
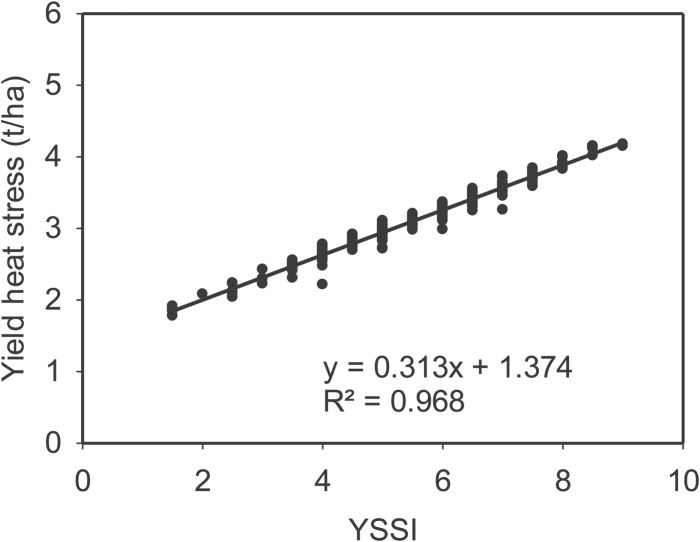
Linear regression and the coefficient of determination of the yield stress score index (YSSI) *versus* grain yield under heat stress conditions (Ys). Calculations use yield data from the WAMI trial (294 genotypes) under heat stress and non-stress conditions during the cropping season 2011–2012.

**Fig. 4.**
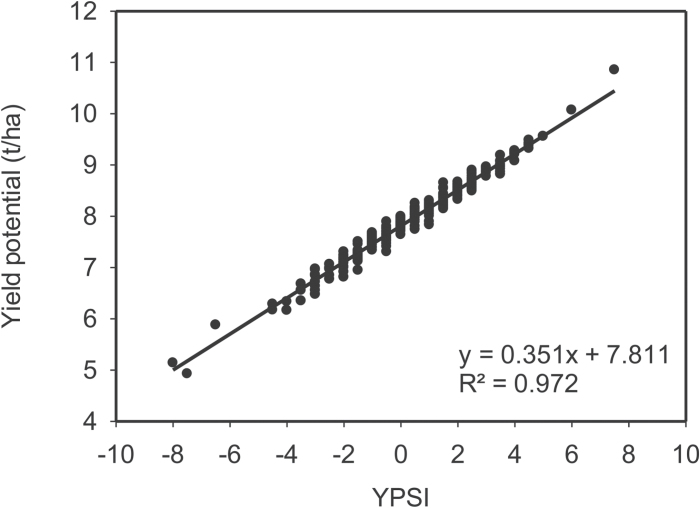
Linear regression and the coefficient of determination of grain yield under a yield potential environment (Yp) and the yield potential score index (YPSI). Calculations use yield data from WAMI trial (294 genotypes) under heat stress and non-stress-conditions during the cropping season 2011–12.

In both equations, the first and second components correspond to PCI and RCI, respectively. The relationship between Ys and Yp and Eqns 1 and 2 is illustrated in Figs 3 and 4, respectively.

These results have demonstrated that yield, either under stress or non-stress, can be expressed by two components, resilience (RCI) and production (PCI). Moreover, the combination of score indices has improved the use of the original indices and their relationship with yield. Additionally, to demonstrate its robustness, this index has been calculated using different multiyear populations (WAMII, Seri/Babax, CIMCOG-ROOT) previously studied in CIMMYT under different abiotic stresses (Pinto *et al.*, 2010; Lopes *et al.*, 2015; Sukumaran *et al.*, 2015). Table 4 shows the Pearson correlation coefficient and coefficient of determination (*R* ^2^) of yield under stress *vs* YSSI. The consistency of the correlations between yield under stress and the index demonstrates the reliability of the index.

**Table 4.** Summary table showing the Pearson correlation coefficient and coefficient of determination (R^2^) of yield score stress index (YSSI) vs yield under stress (Ys) from multiyear populations (WAMII, Seri/Babax, CIMCOG-ROOT) previously studied in CIMMYT under different abiotic stresses (heat, drought, drought under drip, semi-drought (drought applied at booting stage)) The consistency of correlations demonstrates the reliability of the index.

Trial	Entries	Environment	Years	Correlations	*R* ^2^
WAMI	294	Heat	2009–2010	0.988	0.976
WAMI	294	Heat	2010–2011	0.983	0.967
WAMI	294	Heat	2011–2012	0.987	0.975
WAMI	294	Drought	2009–2010	0.987	0.973
Seri/Babax	169	Heat	2004–2005	0.993	0.986
Seri/Babax	169	Heat	2005–2006	0.992	0.984
Seri/Babax	169	Heat	2009–2010	0.955	0.912
Seri/Babax	169	Drought	2005–2006	0.994	0.988
Seri/Babax	169	Drought (drip)	2007–2008	0.989	0.978
Seri/Babax	169	Drought	2008–2009	0.991	0.982
Seri/Babax	169	Drought (drip)	2009–2010	0.974	0.948
CIMCOG ROOT	10	Semi-drought	2012–2013	0.996	0.992
CIMCOG ROOT	10	Heat	2012–2013	0.975	0.95
CIMCOG ROOT	10	Drought	2013–2014	0.981	0.962

In Supplementary Appendix B it is explained why PCI and RCI are complementary and why these combinations work better than the previous indices (SSI, TOL, MP, GMP and STI).

A further combination of the five score indices was named mean score index (MSI):

$$\text{Mean Score Index}=\;\frac{\text{SSIs+TOLs+MPs+GMPs+STIs}}{5}$$ (3)

MSI shows a slightly better correlation with Ys (Supplementary Fig. S1). Nevertheless, this formula contains a disproportion between its productivity and resilience components, giving more weight to the first one with three indices, while containing only two indices for resilience, with the consequent greater impact of the productivity on the output. As the aim of this paper is to identify an easy method to distinguish resilience and productivity, the MSI has not been addressed.

Therefore, YSSI and its components PCI and RCI, which are the scored STI and the scored SSI indices, respectively, are the focus indices of this work in order to improve the selection and identify contrasting genotypes in terms of PC and RC, both under stress conditions.

### How can these indices be used to identify resilient and productive genotypes?

The score indices provide two things. Firstly, the interpretation of yield data collected across large populations of genotypes is much easier as everything is on a similar scale, allowing the visualization of the score (1 to 10) to detect the lowest, medium or highest response. Secondly, the score indices enable us to better understand genotype behaviour under stress, indicating if a high yield under stress is due to tolerance (resilience) or due to a high production capacity (mean yield performance), or both. This can be achieved by analysing the components of YSSI, where high resilient/tolerant and high productive genotypes should have a high value in both indices (RCI and PCI).

Table 5 presents an extended summary of the different indices and their combined value as a function of the values of Yp and Ys. The groups are delimitated by a boundary line (Fig. 1) that represents the minimum or maximum for each group. The boundary line could be represented by the average grain yield within the population under the corresponding environments or by using a local reference line to check yield (both could be used depending on the aim of the research). Consequently, depending on the range of values of Yp and Ys inside the groups, a range of values for RCI and PCI is expected to correspond to the variation of Yp and Ys.

**Table 5.** Summary of the values expected for the different index scales within a population and the impact of their combination as YSSI and YPSI A unique combination of the resilience capacity index (RCI) and the productivity capacity index (PCI) values differentiates the four response groups of plants (A, B, C and D) defined by Fernandez (1992), according to yield stress score index (YSSI) and yield potential score index (YPSI) values. Group A genotypes express uniform superiority in both stress and non-stress condition; group B genotypes express good performance only in yield potential but not under stress conditions; group C genotypes present a relatively higher yield only under stress; group D genotypes present poor yield performance in both environments. Yp: grain yield under yield potential conditions, Ys: grain yield under stress conditions.

Groups	Yp	Ys	RCI	PCI	Combination’s range of values	
					YSSI	YPSI
A	Med-high to high	Med-high to high	Med-high	High	High to med-high	High to med-high
B	Med-high to med	Med-low to low	Med	Med	Med	Med
			Low	Low	Low	Med
				Med	Med-low	Med-high
				High	Med-high	High
C	Med to med-low	Med-high to high	High	Med	High to Med-high	Med to Med-Low
D	Med-low to low	Med-low to low	High	Low	Med-high	Low
			Med		Med-Low	Med-low
			Low		Low	Med-Low

**Fig. 5.**
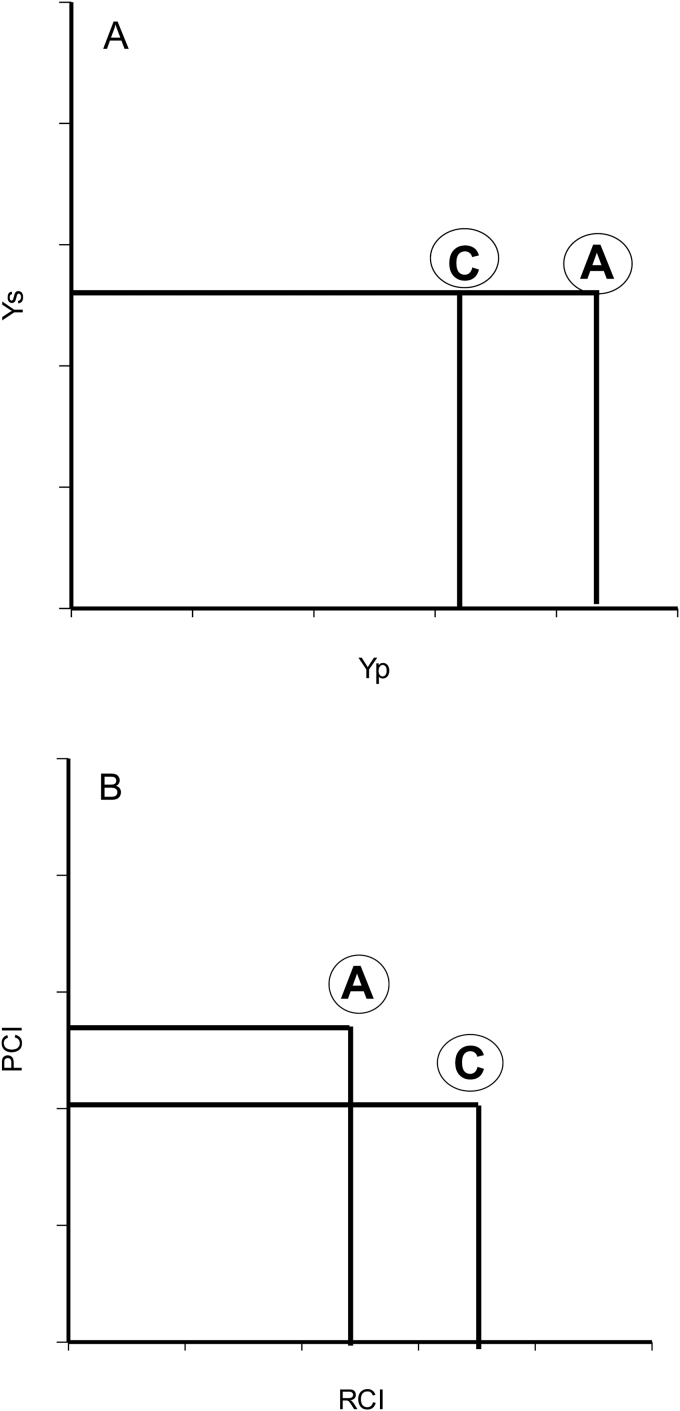
Schematic illustration of a particular case of two genotypes according to grain yield performance defined by Fernandez (1992): A) Schematic illustration of two genotypes from groups A and C with a similar grain yield value under stress (Ys) and different grain yield value under yield potential conditions (Yp), where: group A represents genotypes expressing uniform superiority in both stress and no-stress condition, and group C represents genotypes expressing a relatively higher yield only under stress. B) Schematic representation of the distribution of values of the Productivity Capacity Index (PCI) and the Resilience Capacity Index (RCI) where genotypes A show a higher PCI compared with genotypes C, and *vice versa*, in terms of RCI.

As shown in Table 5, a unique combination of RCI and PCI values identifies and differentiates perfectly the four groups defined by Fernandez (1992); the only case where the combination is not unique is for the low yield under stress that could be obtained from group B or D, both with low PCI and RCI value. These responses could be differentiated using the Yp value, which is higher for genotypes from group B than genotypes from group D. In general, the unique combination and distinction of the different groups is illustrated by an example in Fig. 5, where the resultant value, in this case YSSI, can be similar for genotypes included in groups A and C; however, RCI and PCI will be different between these groups. In this particular case, genotypes from group C show a better resilience (RCI) than genotypes from group A, and genotypes from group A shows a better yield performance under non-stress.

These differentiations can be very useful for a crop breeding programme focused on discovering highly resilient and productive genotypes or only highly resilient ones for crossing with highly productive genotypes. For mechanistic research, contrasting genotypes in terms of resilience or productivity could provide an understanding of the impact of specific trait expression such as stomatal conductance, waxiness and hormone production. For example, high yield production under stress can be derived from a genotype that is tolerant or has a good yield performance under non-stress, or both. Indeed, some genotypes from groups A and C can have a similar yield value under stress conditions, but genotypes from group C will present a lower yield under a non-stress condition (compared with genotypes from group A) but will not reduce much their yield under stress and consequently will have a better resilience to the stress, which can be identified by a higher RCI value. Therefore, the score indices offer the possibility of easily visualizing the plasticity of genotypes in response to a particular stress by looking at the RCI and PCI values. Table 6 shows a simple example for a small trial of ten genotypes under heat stress during the 2012–13 cropping season. One example of a contrasting genotype selection for fundamental research can be taken from these data: genotypes 6 and 1 have a similar YPSI (Yp) but the YSSI (Ys) values are totally opposed, being the highest and the lowest, respectively. Additionally, genotype 6 has the highest PCI and RCI, and genotype 1 shows the lowest index values within the whole population.

**Table 6.**
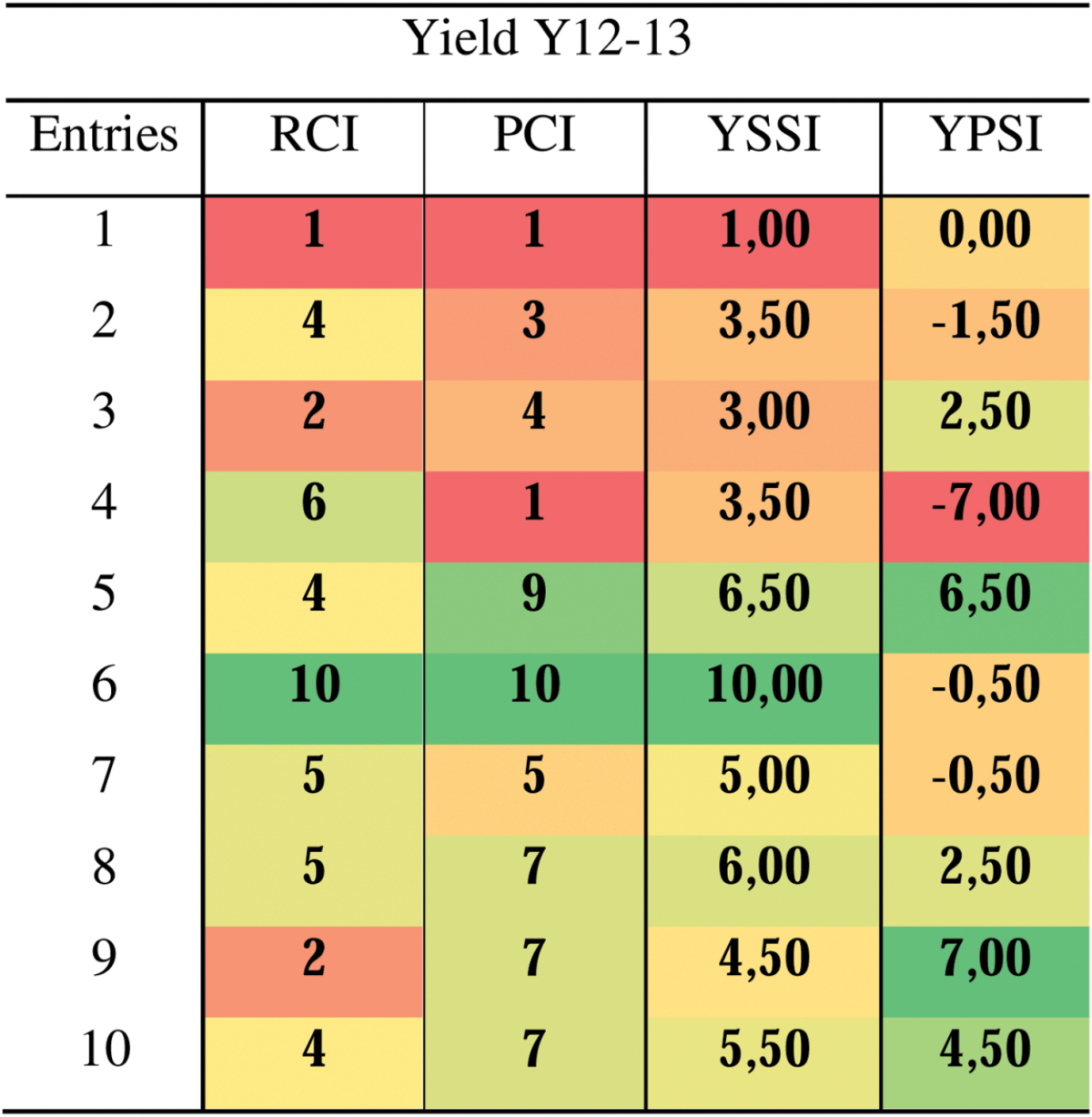
Values of the resilience capacity index (RCI), the productivity capacity index (PCI), and the result of their combination as the yield stress score index (YSSI) and the yield productivity score index (YPSI) Values are calculated using the grain yield data from the CIMCOG-ROOT trial (ten genotypes) under yield potential conditions and heat stress, for the 2012–2013 cropping season (Y12–13).

At this point, an important question has to be raised. Does phenology influence the index selection method? Actually, it is widely known that the ability of plants to recover from abiotic stress (drought or heat) principally depends on the developmental stage at which the plant suffers it (Jäger *et al.*, 2008), or when the stress is applied in cases of indoor experiments. In wheat, meiosis is a stage very sensitive to abiotic stress, which results in reduced pollen fertility and consequently final number of grains (Saini *et al.*, 1984; Acevedo *et al.*, 2006; Jäger *et al.*, 2008). Additionally, Tewolde *et al.* (2006) stated that early-heading genotypes under heat stress had a longer grain ﬁlling period and completed a greater fraction of the grain ﬁlling earlier in the season when air temperatures were lower and generally more favourable compared with the later-heading cultivars. However, early-heading could be considered as an escape strategy instead of a tolerance and/or resilient adaptation. Consequently, in order to improve genotype selection for mechanistic research to discover new traits for stress resilience and to avoid selecting genotypes that may have an escape strategy, phenology should be taken into account. Effectively, as the index compares genotypes within the whole population, early genotypes showing a ‘good’ RCI and/or PCI due to an escape strategy would modify the general range of resilience and/or production capacity with the risk of discriminating some genotypes with late phenology, which actually could present better adaptive/tolerant traits to endure the stress although reducing their yield more, compared with early genotypes, and consequently showing a lower RCI and/or PCI.

Considering this, in order to improve a contrasting selection by integrating the phenology into the index selection method, two procedures can be recommended. The first is, with a small trial with dozens of genotypes, to analyse the whole population and identify separately the early, mid and late genotypes, thus selecting genotypes into groups of similar phenology. For example, in a small trial like CIMCOG-ROOT used for fundamental research, it was observed that genotypes 2 and 4 showed an early phenology, reaching booting stage 5 and 7 days before the late genotypes (9 and 10), respectively, when the mean population reached this stage 3 days before genotypes 9 and 10. Additionally, when genotypes 9 and 10 started meiosis, genotypes 2 and 4 were at the middle of that phase. Two observations can be made from the point of view of the early genotypes. First, genotype 4 is the earliest genotype, starting meiosis 4 days before the population mean, currently behaving as resilient (RCI=6). So, it could present an escape strategy and then turn susceptible, compared with genotypes 7 or 8, if it were to receive the stress at the same phenological stages with the same intensity as genotypes 7 and 8. Second, genotypes 2 and 10 showed similar susceptibility (RCI=4), although genotype 10 received a higher stress during the susceptible phenological stages. Consequently, genotype 10 could be considered more resilient to heat stress than genotype 2 if phenology is taken into consideration. Therefore, in this specific case of the CIMCOG-ROOT trial, genotype 6 seems to be the best to be selected, in terms of adaptation to stress and harvestable yield under stress. However, this genotype will be classified into group C instead of group A, because its Yp is lower than the mean yield of the population. So, selection for abiotic stress tolerance and suitable yield performance should consider genotypes from groups A and C.

The second procedure to integrate phenology into the use of the index for a huge panel would be to analyse the whole population and/or analyse separately the early, mid and late genotypes. For a breeding programme using a selection method based on the proposed index, the problem of different phenology in a panel is similar to the conventional selection on yield, with the only difference that the index method allows us to create ‘new populations’ like the early, mid and late genotypes, and compare each with a reference line to check yield. The more uniform the population is, in terms of phenology, the better the index will perform in identifying contrasting genotypes or genotypes with high yield performance, as one of the bases of the score index method is to compare the genotypes’ response within the whole population. For example, the WAMI trial has been compared between the conventional selection method—based on yield under both environments (stress and no stress)—and (i) the index selection method and (ii) index selection by grouping the genotypes according to their phenology (in this case, heading). For all these cases the selection is done using a reference line (Sokoll) to check yield. The yield method selects genotypes with higher yield under both environments, compared with Sokoll. The index selection refers to the score obtained by Sokoll and selects genotypes with similar score or higher. The first observation is that the index selection reduces the number of selected genotypes by 33%, and the index and phenology method reduces the number of genotypes selected by 48%, compared with the yield check method. The second observation is that with the index method, 64% of genotypes match those of the yield method, being those genotypes classified into group A, while 36% are genotypes that would never have been selected with the conventional method, coming from group C. The third observation comparing the index method and the index integrating the phenology is that late genotypes are generally discarded using the index method.

As mentioned above, the selection methods used by breeders are mainly based on yield production, where genotypes are firstly selected for their ability to produce more yield under both environments, compared with the performance of a local check, and subsequently there is a deeper study in a second phase of selection. However, Blum (1996) has noted that an apparently negative association between yield potential and drought resistance has been found in different studies, where genotypes with a superior adaptation to drought stress may have a lower yield in yield potential environments. However, he specified that this is not always the case, and suggested that the identification of factors involved in the negative relationship will be important for designing a more efficient approach to be used in breeding for high yield and yield stability. We suggest that a wheat improvement programme could beneficially use the score indices, RCI and PCI, simultaneously, in order to identify those rare genotypes that do not show these negative relationships. Such a course of action could reduce considerably the number of selected genotypes, focusing on resilience and productivity, allowing breeders to reduce costs and save time. Finally, the use of a uniform criterion in fundamental research, like RCI and PCI, would ensure more valid and useful comparisons between research results obtained across a selection panel where there is only a hazy understanding of potential selection criteria.

## Conclusions

Score indices offer an easy-to-use new method to classify and visualize quickly which are the best or the worst crop genotypes within a population, in terms of resilience and production. Additionally, score indices allow arithmetic operations to create a new index, YSSI, which expresses yield under stress as a simple score scale value. This expression of yield has demonstrated that yield under stress can usefully be perceived as a function of two major crop characteristics, the resilience capacity and the production capacity.

This analysis provides new insights for selection of genotypes in crop breeding programmes, helping breeders and researchers to understand better the genotypic responses under stress. However, it has been observed that highly productive and highly resilient genotypes (group A) are rare in nature. Therefore, we suggest considering also those genotypes with high resilience (high RCI) and medium-high Yp (high PCI), included in group C. These new indices also offer the opportunity to focus the analysis on resilience to the increasing stress environment in order to improve and assure yield sustainability.

Improvements of this new method could be achieved by grouping the genotypes by their phenology in order to avoid misleading interpretations of resilience when the stress appears at different sensitive phenological stages. Therefore, plants with different phenology (early and late) and similar RCI, calculated within the whole population, could have, in fact, different resilience levels as the early genotypes could show an escape strategy instead of enduring the stress.

## Supplementary data

Supplementary data are available at *JXB* online.

Appendix A. Description of the previous formulas of stress indices.

Appendix B. Explanation of why a combination of PCI and RCI improves the use of the previous indices.

Figure S1. Linear regression and the coefficient of determination of grain yield under heat stress and the mean score index of five stress indices.

Table S1. List of the different score index combinations.

Table S2. Correlation between index combinations and grain yield under yield potential and under heat conditions on CIMCOG-ROOT trial.

Table S3. Correlation between index combinations and grain yield under yield potential and under drought conditions on CIMCOG-ROOT trial.

Table S4. Correlation between index combinations and grain yield under yield potential and under heat conditions on WAMI trial.

Supplementary Data

## Abbreviations:

CIMCOG: CIMMYT core germplasm
GMP: geometric mean productivity index
GMPs: score geometric mean productivity index
MP: mean productivity index
MPs: score mean productivity index
MSI: mean score index
PC: production capacity
PCI: production capacity index
PT: physiological trait
RC: resilience capacity
RCI: resilience capacity index
SSI: stress susceptibility index
SSIs: score stress susceptibility index
STI: stress tolerance index
STIs: score stress tolerance index
TOL: tolerance index
TOLs: score tolerance index
WAMI: Wheat Association Mapping Initiative trial
Yp: grain yield under yield potential conditions
YPSI: yield potential score index
Ys: grain yield under abiotic stress conditions
YSSI: yield stress score index
