# Supplementary material for: How can we improve crop genotypes to increase stress resilience and productivity in a future climate? A new crop screening method based on productivity and resistance to abiotic stress
Source: J Exp Bot. 2016 Sep 27;67(19):5593–603. doi: 10.1093/jxb/erw330 (PMC5066489; doi:10.1093/jxb/erw330)
Supplement: Supplementary Data [file supp_67_19_5593__index.html]

How can we improve crop genotypes to increase stress resilience and productivity in a future climate? A new crop screening method based on productivity and resistance to abiotic stress — How can we improve crop genotypes to increase stress resilience and productivity in a future climate? A new crop screening method based on productivity and resistance to abiotic stress — Supplementary Data 

# How can we improve crop genotypes to increase stress resilience and productivity in a future climate? A new crop screening method based on productivity and resistance to abiotic stress

## Supplementary Data

Data files

- Appendices\_A\_and\_B\_Supplementary\_figures\_S1\_supplementary\_tables\_S1\_S4.pdf - Supplementary Data
