# Supplementary material for: How can we improve crop genotypes to increase stress resilience and productivity in a future climate? A new crop screening method based on productivity and resistance to abiotic stress
Source: J Exp Bot. 2016 Sep 27;67(19):5593–603. doi: 10.1093/jxb/erw330 (PMC5066489; doi:10.1093/jxb/erw330)
Supplement: Supplementary Data [file supp_erw330_Appendices_A_and_B_Supplementary_figures_S1_supplementary_tables_S1_S4.pdf]

Appendix A

Description of the previous formula of Stress indices

The Stress Susceptibility Index (SSI) by Fisher and Maurer (1978) is expressed by the following relationship:

$$SSI = \frac{1-\frac{Ys}{Yp}}{SI} \text{ Equation S1}$$

Ys = Yield under stress condition

Yp = Yield under yield potential condition

SI = stress intensity expressed in turn by

$$SI = \left[1 - \frac{\overline{Ys}}{\overline{Yp}}\right] \text{ Equation S2}$$

Where: $\overline{Ys}$ = mean yields overall population under stress condition;

$\overline{Yp}$= mean yields overall population under yield potential condition.

A greater stress tolerance is shown by smaller values of SSI. However, this index fails to distinguish groups A and C (Table 1), owing to the fact that it favours genotypes with medium yield under yield potential and high yield under stress conditions.

Rosielle & Hamblin (1981) created the stress tolerance (TOL) index, which can be explained as the difference between Yp (yield potential) and Ys (yield under stress):

$$TOL = Yp - Ys \text{ Equation S3}$$

The high value of TOL indicates a low stress tolerance. However, as with SSI, it fails in favouring those genotypes with high yield under stress and low yield under normal conditions. As a result, it cannot discriminate between groups A and C.

Rosielle & Hamblin (1981) also proposed the mean production index (MP) by averaging Ys and Yp (Equation S4.), where a high stress tolerance is indicated by a high MP value. However, it fails in favouring genotypes with high yield under yield potential and lower yield under stress. As a consequence, it cannot differentiate between group A and group B (Table 1).

$$MP = \frac{Ys+Yp}{2}$$ **Equation S4**

Fernandez (1992) explains that the arithmetic mean used in the calculation of MP presents a bias due to a relative larger difference between Yp and Ys. On the contrary, the geometric mean is less sensitive to large extreme values. Thus, he suggested the geometric mean productivity index (GMP) as a better one to distinguish group A.

$$GMP = \sqrt{Ys\,.Yp}$$ **Equation S5**

Fernandez (1992) has also defined another stress tolerance index (STI) with the purpose of identifying genotypes that produce high yield under both environments (yield potential and stress conditions) (Group A). It is expressed by the following relationship:

$$STI = \frac{Yp}{\overline{Yp}}\frac{Ys}{\overline{Ys}}\frac{\overline{Ys}}{\overline{Yp}} = \frac{Yp\,.\,Ys}{\overline{Yp}^2}$$ **Equation S6**

The higher value of GMP and STI represents a higher tolerance to stress.

Appendix B

Explanation of why the combination of PCI and RCI improve the use of the previous indices

The high correlation of YSSI and YPSI with grain yield (Ys and Yp, respectively) can be explained due to the readjustment of the PCI and RCI values of the different groups defined by Fernandez (1992), where group A) genotypes express uniform superiority in both stress and no-stress condition; group B) genotypes express good performance only in yield potential but not under stress conditions; group C) genotype presents a relatively higher yield only under stress and group D) poor yield performance in both environments. Fig. B1 illustrates the position of the different groups, as a function of Yp or Ys, *versus* PCI or RCI. The position of groups B and C are the only ones to change when the same index is looking at Yp or Ys. This explains why it has been observed a good relationship among the previous indices and both yields (Yp and Ys), since half of the data (groups A and D) keep the same range of values, where group A has the highest value and group D the lowest value.

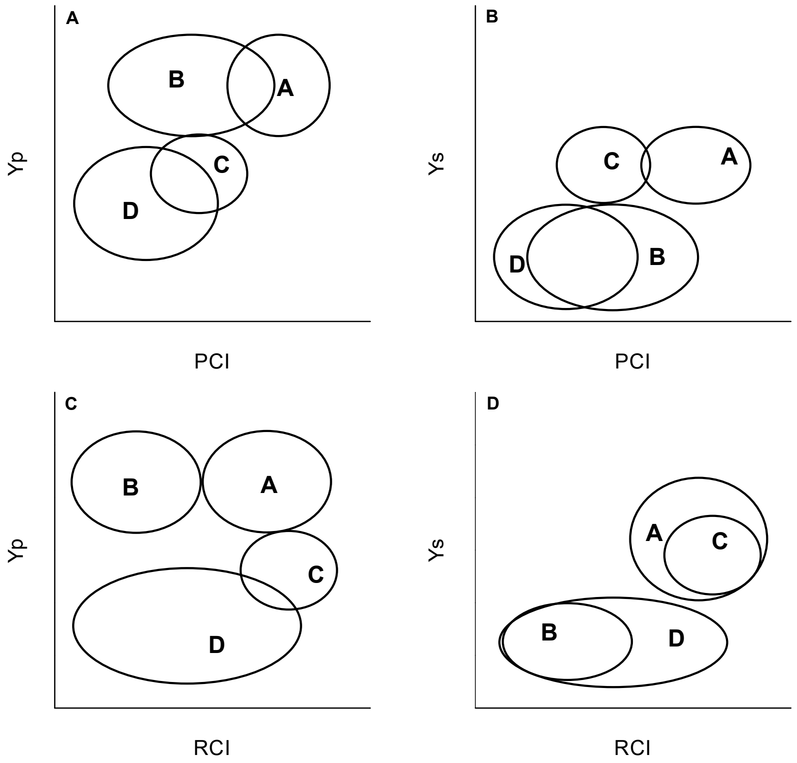

Figure B1: Schematic illustration of the position of the different groups defined by Fernandez (1992) as a function of Yp or Ys *versus* PCI or RCI: (A) PCI *versus* Yp; (B) PCI *versus* Ys; (C) RCI *versus* Yp, and (D) RCI *versus* Ys.

Thus, the combination of both score indices (RCI and PCI) re-arranges the groups according to grain yield (Yp or Ys) following a linear response (Fig. B2).

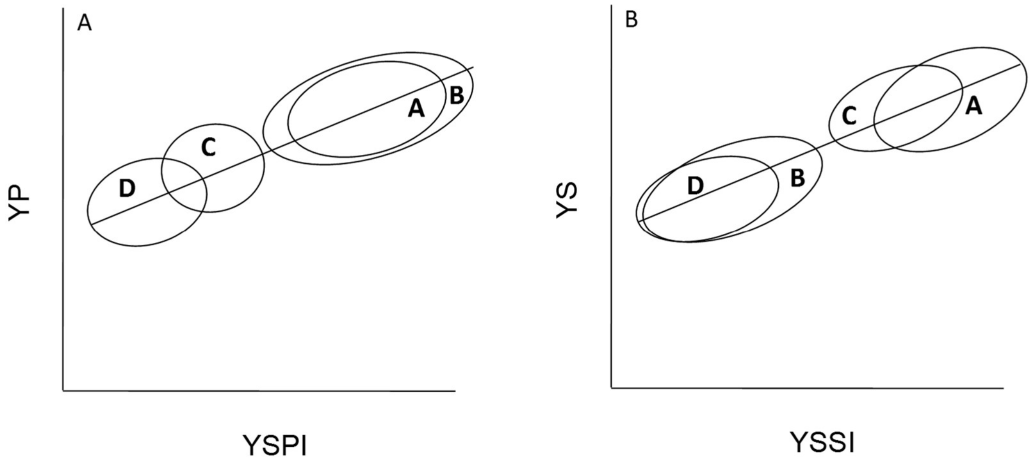

Figure B2: Schematic illustration of the classification of the different groups defined by Fernandez (1992) over the linear regression (A) Yp *versus* YPSI (B) Ys *versus* YSSI.

To understand better YSSI, a particular case, where a similar Ys could be obtained by genotypes producing more or less Yp (groups A and C) (Fernandez, 1992), is studied. In this case, a genotype from A will have a higher PCI, due to a higher Yp, and a lower RCI, due to a higher reduction of yield compared with a genotype from C. Thus, the combination of a high PCI and a low RCI (genotype A) will give a similar value compared with the combination of a low PCI and a high RCI (genotype C). Then, for a similar Ys, the genotype from C will be more resilient than the genotype from A.

To understand YPSI, the interpretation is slightly different, as two genotypes from groups A and B with a similar high Yp will have different Ys. In terms of PCI and RCI, the genotype from A will always have a higher value than the genotype from B (Fig. B3). Thus, the genotype B has a lower PCI value due to its low yield under stress. Therefore, if the susceptibility is removed from the mean production (PCI) of genotypes A and B, it is obtained a similar value which is correlated with Yp.

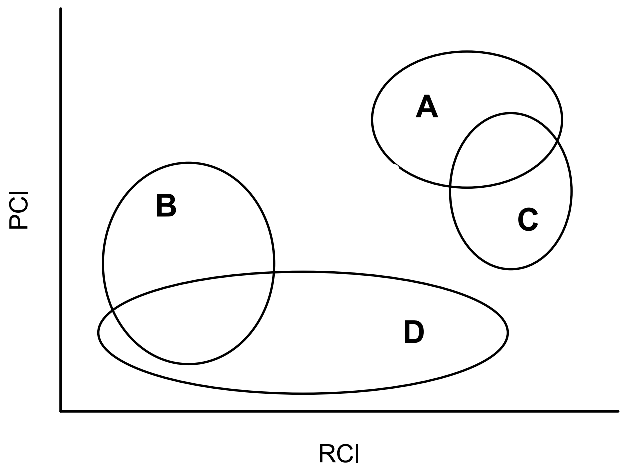

Figure B3: Schematic illustration of the different values of PCI and RCI of different groups according to Fernandez (1992), showing a small area where groups A and C could not be differentiated, and similarly for B and D.

Supplementary figure

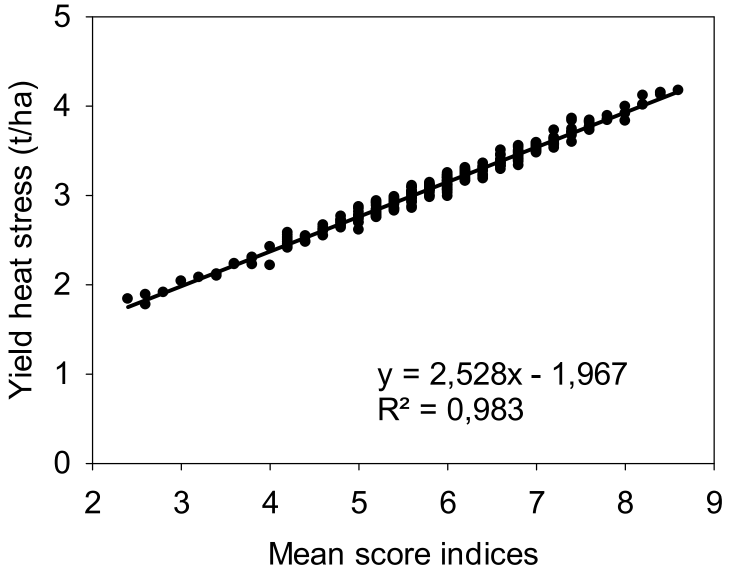

Figure S1: Linear regression and the coefficient of determination of grain yield under heat stress (Ys) and the mean score index. Calculations of the score indices use yield data from WAMI trial (294 genotypes) under heat stress and non-stress-conditions during the cropping season 2011-12.

Supplementary tables

Table S1: List of the different score index combinations assessed to identify the best correlation with grain yield under yield potential and stress conditions.

Addition	Subtraction
**By pair**	
$\frac{(\mathrm{SSIs+MPs})}{2}$ Combination 1	$\frac{(\mathrm{MPs-SSIs})}{2}$ Combination 7
$\frac{(\mathrm{SSIs+GMPs})}{2}$ Combination 2	$\frac{(\mathrm{GMPs-SSIs})}{2}$ Combination 8
$\frac{(\mathrm{SSIs+STIs})}{2}$ Combination 3	$\frac{(\mathrm{STIs-SSIs})}{2}$ Combination 9
$\frac{(\mathrm{TOLs+MPs})}{2}$ Combination 4	$\frac{(\mathrm{MPs-TOLs})}{2}$ Combination 10
$\frac{(\mathrm{TOLs+GMPs})}{2}$ Combination 5	$\frac{(\mathrm{GMPs-TOLs})}{2}$ Combination 11
$\frac{(\mathrm{TOLs+STIs})}{2}$ Combination 6	$\frac{(\mathrm{STIs-TOLs})}{2}$ Combination 12
**By group**	
$\left(\frac{(\mathrm{SSIs+TOLs})}{2}+\frac{(\mathrm{MPs+GMPs})}{2}\right)$ Combination 13	$\left(\frac{(\mathrm{MPs+GMPs})}{2}-\frac{(\mathrm{SSIs+TOLs})}{2}\right)$ Combination 16
$\left(\frac{(\mathrm{SSIs+TOLs})}{2}+\frac{(\mathrm{MPs+STIs})}{2}\right)$ Combination 14	$\left(\frac{(\mathrm{MPs+STIs})}{2}-\frac{(\mathrm{SSIs+TOLs})}{2}\right)$ Combination 17
$\left(\frac{(\mathrm{SSIs+TOL\ })}{2}+\frac{(\mathrm{GMPs+STIs})}{2}\right)$ Combination 15	$\left(\frac{(\mathrm{GMPs+STIs})}{2}-\frac{(\mathrm{SSIs+TOL\ })}{2}\right)$ Combination 18

Table S2: Pearson coefficient between index combinations and grain yield under yield potential and under heat conditions during the cropping season 2012-13 on the CIMCOG-root trial (10 genotypes).

Combinations	Yp	Ys
1	0.411	0.976
2	0.258	**0.997**
3	0.258	**0.997**
4	0.017	0.976
5	-0.121	0.937
6	-0.121	0.937
7	0.983	0.055
8	0.989	0.233
9	0.989	0.233
10	0.995	0.171
11	0.993	0.293
12	0.993	0.293
13	-0.146	0.932
14	-0.146	0.932
15	-0.183	0.917
16	**0.997**	0.193
17	**0.997**	0.193
18	0.994	0.271

Table S3: Pearson coefficient between index combinations and grain yield under yield potential and yield under drought conditions for the cropping season 2013-14 on the CIMCOG-root trial (10 genotypes).

Combinations	Yp	Ys
1	0.273	0.940
2	0.002	0.994
3	0.001	0.996
4	0.007	0.983
5	-0.277	0.959
6	-0.258	0.959
7	0.980	-0.223
8	0.992	-0.018
9	0.987	0.031
10	0.997	-0.004
11	0.968	0.201
12	0.958	0.239
13	-0.520	0.873
14	-0.510	0.878
15	-0.523	0.870
16	0.998	-0.010
17	0.997	0.011
18	0.983	0.125

Table S4: Pearson coefficient between index combinations and grain yield under yield potential and heat conditions during the cropping season 2012-13 on the WAMI trial (294 genotypes).

Combinations	Yp	Ys
1	0.348	0.980
2	0.247	0.983
3	0.258	**0.984**
4	0.173	0.941
5	0.073	0.941
6	0.094	0.949
7	0.896	-0.062
8	0.954	0.198
9	0.960	0.222
10	0.985	0.299
11	0.962	0.512
12	0.960	0.522
13	-0.110	0.889
14	-0.103	0.892
15	-0.110	0.892
16	0.985	0.259
17	**0.986**	0.269
18	0.986	0.394
